# Medical Graduates

**Published:** 1878-05-20

**Authors:** 


### MEDICAL GRADUATES.

The aggregate number of graduates in medicine in the United States, for the year 1878, is 1,723. Of these, there graduated in the Northern schools, 783, Western schools, 656; Southern schools, 284—Augusta, Charleston and New Orleans not included.

### POCKET DRUG CASES.

One of the most beautiful pocket drug cases we have ever seen, is that manufactured by Wm. R. Warner & Co., of Philakelphia. It contains ten 2 oz. phials, neatly labeled. A sample has been kindly sent us by the eenergetic and indefatigable gentlemen of this large and splendid establishment, whose skill, taste and energy is evinced in all the varied departments of their extensive home and foreign business.

### S. M. PETTINGILL & CO.'S NEWSPAPER DIRECTORY AND HAND-BOOK OF REFERENCE.

This is a very valuable and useful book. It is neatly gotten up, is complete in its arrangements, and contains information from nearly nine thousand papers and periodicals, so arranged as to be readily found, and so reliable that advertisers may consult it with confidence. This cannot be said of every other directory in print.

---

Park, Davis & Co., whose advertisement may be seen on first inside page of this Journal, have forwarded us samples of their preperations. Among which are a number of new medicines. They are put up in a very neat and careful manner. This house is certainly managed by very capable and reliable men. To discribe the various articles sent us as they evidently merit, would draw too heavily on our space. Hope to bring them before the profession from time to time in communicating the advances in therepeutics. Those who desire to test the new agents should communicate with the Proprietors.

### TROMER EXT. OF MALT COMPANY.

The malt preparations of the above reliable company are of unquestioned excellence. The varied combinations of the malt which they prepare are admirable, and are variously ccmbined, so as to meet a great variety of therepeutic indications.

---

Practitioner's Reference Book.—See advertisement of this valuable work. It contains highly useful and important information for the practitioner. The premium offer which we make will enable those who avail themselves of it to get the work at a very low figure.

